# ‘You know where we are if you need us.’ The role of primary care in supporting patients following pancreaticoduodenectomy for cancer: a qualitative study

**DOI:** 10.3399/BJGPO.2021.0154

**Published:** 2022-04-06

**Authors:** Anna Kathryn Taylor, Ambareen Kausar, David Chang, Alison Phelan, Carolyn Anne Chew-Graham

**Affiliations:** 1 School of Medicine, Leeds Institute of Health Sciences, Faculty of Medicine and Health, University of Leeds, Leeds, UK; 2 Department of General Surgery, Royal Blackburn Hospital, East Lancashire Hospitals NHS Trust, Blackburn, UK; 3 Patient Advisory Group Member, Blackburn, Lancashire, United Kingdom; 4 School of Medicine, Keele University, Keele, UK

**Keywords:** primary health care, general practice, pancreatic neoplasms, qualitative research

## Abstract

**Background:**

Ten per cent of patients diagnosed with pancreatic cancer undergo pancreaticoduodenectomy. It is known that these patients have unmet psychological support needs, and GPs are key in enabling effective coordination of care for people living with life-shortening conditions.

**Aim:**

To explore patients’ perspectives on the role of primary care in their management, and their sources of support.

**Design & setting:**

Inductive qualitative study of patients who had undergone pancreaticoduodenectomy between 6 months and 6 years previously for pancreatic or distal biliary duct cancers. Participants were recruited by clinical nurse specialists (CNSs) from a single NHS trust in Northwest England.

**Method:**

Semi-structured interviews, either face-to-face or via video link, were conducted with 20 participants. Interviews were audio-recorded, transcribed, and anonymised. Thematic analysis utilised principles of constant comparison.

**Results:**

Participants described immense treatment burden and uncertainty around the role of the GP in their ongoing care. They recognised that GPs may have little experience of patients who have undergone pancreaticoduodenectomy, but felt that GPs can play a vital role in offering support. Participants wished for emotional support postoperatively, and valued support networks including family and friends. However, they found expressing their deepest fears difficult. Participants felt they would value greater recognition by primary care of both physical and psychological sequelae of major pancreatic surgery, and the impact on their families.

**Conclusion:**

Patients may feel themselves to be a ‘burden’ to both healthcare professionals and their own support networks following pancreaticoduodenectomy. Primary care is in a key position to proactively offer psychological support.

## How this fits in

Pancreatic cancer is distinct from other cancers owing to its high mortality and limited treatment options. Patients undergoing pancreaticoduodenectomy have high levels of unmet need. There has been limited qualitative research exploring the experience of support-seeking by these patients and how they perceive the role of primary care. This study makes explicit that patients identify their GP as a key potential source of psychological support but they frequently lack the confidence to seek out their GP for such support.

## Introduction

Pancreatic cancer is the 10th most common cancer in the UK,^1^ with most diagnosed after metastasis.^2^ However, approximately 10% of patients diagnosed with pancreatic and biliary duct cancers have surgery, with or without chemotherapy or radiotherapy.^3^ The most common type of surgery performed for pancreatic adenocarcinoma, distal cholangiocarcinoma, and periampullary tumours is a pancreaticoduodenectomy (or Whipple’s procedure).

There is limited research focusing on the experiences of patients living with pancreatic cancer; most qualitative studies have focused on decisionmaking around treatment,^4,5^ secondary care surveillance,^6,7^ and symptom appraisal before diagnosis.^8,9^ However, it is known that patients who undergo pancreaticoduodenectomy owing to adenocarcinoma report lower quality of life.^10^ Half the participants in a 2019 cross-sectional study reported that they had at least one moderate or high unmet need, such as anxiety, uncertainty, or fear.^11^ People aged >70 years may experience high levels of distress persisting for 5 years after cancer diagnosis;^12^ since the incidence of pancreatic cancer is strongly related to age,^1^ recognising and managing psychological distress is vital.

Patients with pancreatic cancer usually develop pancreatic exocrine insufficiency, which leads to malabsorption and symptoms such as steatorrhoea, bloating, cramping, and weight loss.^13^ This often persists following surgery owing to the volume of pancreas removed. Patients report that these gastrointestinal symptoms and dietary changes significantly impact on their physical, social, and emotional wellbeing.^14–16^ Pancreatic enzyme replacement therapy (PERT) has been shown to significantly improve fat digestion and reduce symptoms of malabsorption, with few side effects reported.^17,18^ Prescription of PERT (such as Creon) is now standard practice in patients with pancreatic cancer and is done in primary care.^19,20^

GPs are crucial in enabling the effective coordination of care for patients living with life-limiting conditions, and identifying unmet support needs that negatively impact on patients’ lives. However, it is often unclear whether the responsibility for care of those living with and beyond cancer lies with primary, secondary, or tertiary care.^21^ A large cross-sectional survey found that 59% of patients with a previous cancer expressed a need for increased GP involvement in cancer care, with the majority also stating that GPs are well-placed to listen to patients’ concerns and discuss their priorities in order to support shared decisionmaking.^22^ Patients who perceive the GP to be informed about their cancer were more satisfied with treatment decisions,^23^ and those who speak with their GPs between diagnosis and the start of treatment have improved satisfaction.^24^ Satisfaction with GP involvement has been shown to be higher if the GP is the initiator of contact,^22,25^ but GPs may be reluctant or unable to do this owing to lack of time and perceived lack of knowledge of, and expertise in, the specific cancer.^26,27^ A systematic review of the views of patients on the role of the GP highlighted that patients desire a biopsychosocial approach and want GPs to be better engaged in cancer care.^28^

There has been little exploration of patients’ perceptions of the role of the GP in the provision of ongoing care of patients following a Whipple’s procedure for cancer. Similarly, neither the burden of ongoing recovery nor other sources of support sought by such patients have been examined.

## Method

Qualitative methodology was used to explore the perspectives of patients about their care following pancreaticoduodenectomy for cancer. Semi-structured interviews allowed participants to talk about areas they felt were important, while ensuring all topics were covered.^29,30^ This article conforms to appropriate qualitative reporting guidelines.^31^

CNSs identified potential participants currently under the care of a tertiary hepatopancreaticobiliary (HPB) centre in northwest England. Inclusion criteria were if they had had pancreaticoduodenectomy 6 months to 6 years previously for either head of pancreas cancer, distal cholangiocarcinoma, or periampullary cancer, and had completed chemotherapy. Patients were excluded if they were aged <18 years, if they had a current diagnosis of a severe mental illness (determined through review of GP-coded diagnoses), lacked capacity to consent, did not speak sufficient English, or were in their last days of life. Fifty-two patients met these criteria. All participants had completed active treatment following diagnosis (surgery with/without chemotherapy), although some had had recurrence since.

A random number generator selected five patients at a time. They were telephoned by a CNS and told about the study; if they consented to further contact, they were emailed or posted the participant information sheet and invited to contact the researcher conducting the interviews (AKT).

Between December 2019 and February 2020, 16 interviews were conducted at the hospital or in participants' homes, depending on their preferences. Recruitment was halted owing to COVID-19 restrictions and then restarted in July 2020, with four interviews being conducted via a virtual video platform.

Before the interview, participants gave written consent to participate and for audio-recording. The topic guide was developed using the existing literature and with input from a patient advisory group (PAG), and was used flexibly with open questioning to generate data. Interviews explored participants’ experience of surgery and chemotherapy, the impact of cancer on their life, sources of support, and access to primary and secondary care. No time limit was imposed on the interview and all came to a natural end. Participants were offered a £20 gift card to thank them for their time.

Following recording, interviews were transcribed verbatim and anonymised. The research team included one junior doctor (AKT), two HPB surgeons (DC and AK), one member of the PAG (AP), and one GP (CCG). Line-by-line coding and inductive thematic analysis of all transcripts^32^ was undertaken by one researcher (AKT), and the other researchers (AK, DC, AP, CCG) each analysed a subset of transcripts. Researchers (AK and DC) did not analyse or see the transcripts of any patients whose care they were directly responsible for, in order to avoid bias. Codes were discussed collaboratively to identify and agree key themes, and the analysis was continually refined using the principles of constant comparison.^33–35^ Good agreement and triangulation of themes and codes was achieved. Data saturation, the point at which no new themes were derived,^36^ was reached at 18 interviews; two further interviews were undertaken to confirm this before recruitment was stopped.

### Patient and public involvement and engagement

A patient advisory group (PAG) was convened to discuss the aims and methods of the study with patients who had undergone pancreaticoduodenectomy at least 6 years previously and were thus ineligible for inclusion in the study. PAG members commented on the topic guide, invitation letter, and participant information sheet, all of which were refined to reflect their suggestions. The PAG was reconvened following completion of the interviews to discuss preliminary analysis, invite reflection, and suggest dissemination strategies. One member of the PAG joined the authorship team to engage in further analysis and writing. Finally, a lay summary was circulated to participants following completion of analysis.

## Results

Twenty participants were interviewed (demographics reported in Table 1). Interviews lasted between 51 and 187 minutes (mean 105 minutes). Four patients approached declined to be interviewed, stating they felt that reflecting on their cancer diagnosis would distress them; 11 who were eligible for inclusion at the start of the recruitment period died before they could be invited to participate. One eligible patient developed a new primary malignancy during the recruitment period and was therefore not invited to participate.

**Table 1.** Participant demographics (*n* = 20)

Characteristic	Frequency,*n* (%) unless otherwise stated	Median, IQR
Female	10 (50.0)	
White British	20 (100.0)	
Tertiary referral	12 (60.0)	
Mean age at diagnosis (range)	65.2 years (45–79 years)	66, IQR 14.8
Mean age at interview (range)	67.9 years (47–82 years)	68.5, IQR 16.5
Mean months since surgery (range)	24.5 months (10–72 months)	20.5, IQR 13.0
Mean length of hospital stay (range)	22.1 days (7–96 days)	15.5, IQR 16.5
Chemotherapy before surgery	1 (5.0)	
Chemotherapy after surgery	16 (80.0)	
Recurrence	4 (20.0)	
Initial presentation as emergency	10 (50.0)	

IQR = interquartile range.

The following themes will be presented in this article, with illustrative quotes identified by a pseudonym for each participant: feeling a burden and being burdened; sources of support; and what I would like my practice to do.

### Feeling a burden and being burdened

The majority of participants reflected that following treatment they often felt unsure about who to approach if they had questions, and did not seek help from their GP:

*‘After my Whipple I remember thinking “I don’t really know where I stand now, whether I see my GP or the oncologist or* [surgeon]*’’.’* (Helen)

Participants recognised that GPs may have little experience managing patients who had had a pancreaticoduodenectomy for cancer, and these opinions were sometimes compounded by negative comments from secondary care about their GP’s knowledge, which made help-seeking difficult:

*‘She doesn’t seem to know anything about my condition, that’s the trouble. They probably haven’t got another patient that’s had a Whipple’s procedure.’* (Margaret)

*‘The specialist nurses said to me, “Your GP surgery won’t even know anybody that’s had a Whipple, it’s a very big operation, you’ll be lucky if they’ve had a patient that’s had it.” Which I found a bit upsetting, I thought well how are they supposed to look after me?’* (Janet)

Some participants felt that sometimes the burden had been placed on them to educate their GP about aspects of their care:

*‘She’s OK but she’s not one of the best GPs, and essentially whatever I ask she does, she’s never suggested anything. The thing is, I know more about it than they do. There’s no getting away from it.’* (Alice)

*‘It’s been written down that you take two* [PERT] *three times a day, and, but that is no good because say I went out and had fish and chips I would need at least five. So they give me 300 a month, but sometimes 300’s not enough. So I’ll put on my repeat prescription, please ask the doctor if I can have 400 this month. No, I get 300. And I said, “Do you not read what I’ve written?” “But your thing says two three times a day” and they still put that on my tablets and that’s rubbish.’* (Theresa)

In addition to feeling a burden when help-seeking, many participants felt that they were a burden on their family, feeling guilty for being unable to support them while were undergoing treatment or dealing with recurrence:

*‘*[My wife] *was a brick for me. She was … I’ve tried to make it up since ... I used to apologise to her and say, “I really hate this,” but she said, “Well you’d do it for me if I was the same”… We’ve been through it together. And I couldn’t imagine it without her.’* (Joseph)

*‘My wife is quite capable of coping when I’m gone but I apologised to her, I said, “I’m so sorry,” and she said, “What are you sorry for?” and I said, “Because I’m gonna be leaving you.”’* (Thomas)

Participants also felt the burden of managing their new medication, particularly PERT and insulin:

*‘I’m injecting the basal insulin, I’m injecting that twice a day, and the other insulin, I’ve to inject that every time I have food but before I can eat I’ve got to work out how many carbs there are in the food and then assess how much insulin I need … life is just hard, it really really is hard.’* (Margaret)

*‘They ran out of the 25 000 so they put me on 10 000 but that meant that instead of taking six with a meal I had to take 15 … So I’d be going through a pack of 100 in two days … it’s a lot of tablets to take … but now we’ve got the normal tablets again it’s easier.’* (Raymond)

*‘Although he said that I would be on tablets for the rest of my life I somehow thought that they would be like my husband’s metformin, you know one in the morning and one at night.’* (Mary)

Others felt that their medications were a visible and troubling reminder of their illness:

*‘I’m reminded of it every meal when I take Creon. Every morning I have lansoprazole. I was hoping I could come off drugs altogether and further put it behind me, but that isn’t going to happen.’* (Tim)

### Sources of support

Participants reported seeking support from a variety of sources including family, friends, and faith communities:

*‘It’s been tough for her as it has for me, but we’ve hacked through it together … I’ve got somebody to talk to and my wife will keep me in check, “have you done this, have you done that.” She’s fantastic. So that is the major part of it. I would feel for somebody who didn’t have that kind of backup. Even when people are telling you things, it’s nice to have somebody else listening ‘cos at the critical stages so much is flying over your head you cannot possibly take it all in, but if you’ve got a second person there it helps.’* (Frank)

*‘I had a lot of support from people at church … we’re like a community you know … that makes me feel a lot better because I’ve got friends there.’* (Margaret)

Although participants identified these sources as supportive, there were often tensions in their accounts. They reflected that they did not share their deepest fears and anxieties, not wanting to upset others, and frequently worried about the impact of their illness on their family. Some highlighted the lack of support for family members of people with cancer:

*‘I feel guilty for this. Because it’s me that’s caused this … Because it doesn’t just affect you, it affects all your family … I don’t want to talk about it to them … ‘Cos we’re really close, you know. They rely on me emotionally … My daughter and granddaughters knew I had cancer but … if I broke down in front of them then they would get upset.’* (Theresa)

*‘*[My husband] *doesn’t want to talk about it … I have tried. And I think he thinks … it’ll come back if we discuss it … My fear in that way is that if I had a recurrence I don’t know how he would cope, this time around. Having been through it once ... And nobody asks how he is. Carers are going through it just as much if not more, because they’ve got to try and be strong for everybody*.’ (Helen)

Many participants were aware of support groups, either online or in person, but most had not engaged with them consistently or found them beneficial:

*‘I did actually sign up for a couple* [of online groups] *but I found it a bit depressing … All people saying such-a-body died on this date 12 months ago and stuff like that. I thought “I don’t need this” so I came out of them.’* (James)

### What I would like my practice to do

Participants felt that their general practice should be proactive in offering both physical and psychological support, and share the burden of being a patient with them:

*‘We thought the doctor would come round to see me after I was discharged. And in the end my husband sent for the doctor, said, “Why has nobody come to see her or anything?” And he did come … but he just said, “You know where we are if you need us,” and that were it, sort of thing … You should have more support from medical people.’* (Margaret)

Some participants highlighted that a lack of continuity of care made it more challenging for them to seek help from someone they felt knew them:

*‘I’ve been with that surgery, though they’re all different doctors now, from being a baby. And some of the good doctors what were there in the last 10 years have all left … You can’t get a blooming appointment and that. And every time you go there was somebody different*.’ (Patricia)

Most participants felt that their GP should play a key role in supporting their ongoing physical and psychological care. A minority reported that they had regular follow-up instigated by the same GP and had been asked about their psychological recovery. These participants said they felt more confident about being able to approach their GP if they did have concerns or needed support, and considered their GP a vital person in their support network:

*‘One of the senior GPs rings me once every couple of weeks and says, “Are you OK to talk for ten minutes?” And he asks me if I need anything or if there’s anything I want ... they’ve said anything I need whether it’s support, somebody to chat to, a doctor to talk to, don’t hesitate to ring them.’* (Thomas)

*‘I was in there a long time, more than the five or ten minutes you’re allowed, you know. And he was very supportive and he said, “My door is always open”… I was very depressed at one time … And I would see him quite a lot, it was like once a month. He’s a doctor that you can talk to.’* (Elizabeth)

## Discussion

### Summary

The accounts of the participants have illustrated not only the burden of being a patient in terms of the treatment itself, but also the burden of having a less-common cancer with unusual and complex surgical treatment. Many participants also felt guilt, perceiving themselves as a burden on their family. Participants sought support from family, friends, and faith communities but reflected that they often felt unable to share their deepest worries, and were often concerned about the impact of their illness on their family. Few participants had found support groups helpful.

Participants felt that their GP practice should proactively offer physical and psychological support in their cancer journey. Lack of continuity of care was cited as a barrier to support-seeking. A minority had had regular follow-up prompted by the GP, and these participants felt more confident in approaching their GP if they had concerns or needed support.

### Strengths and limitations

This study is, to the authors' knowledge, the first to explore the sources of support and the role of primary care for patients following pancreaticoduodenectomy for cancer. Semi-structured interviews enabled participants to speak in depth about experiences that they felt were important, while ensuring all aspects of the topic guide were covered. The researcher conducting the interviews (AKT) was not involved in any of the participants’ direct patient care, which mitigated bias. The sample included participants of a range of ages and at a range of points in their journey, including those who had only recently completed adjuvant chemotherapy and those with recurrence. Bias was also mitigated through independent coding, and there was good agreement between researchers when refining themes. It is a particular strength that the authors have differing backgrounds, including surgery, primary care, psychiatry, and lived experience of pancreatic cancer. This, along with the perspectives of members of the PAG, enabled richer exploration of the data.^37^

A limitation is that the findings from this single-centre qualitative study may not extrapolate to other geographical areas, particularly given a lack of racial diversity.

### Comparison with existing literature

There is limited research focusing specifically on the role of primary care in supporting patients with pancreatic cancer. The participants often felt unsure who to approach if they had questions or concerns, and, for some, this uncertainty was exacerbated by dismissive comments made by hospital staff about how GPs could help. Participants described the hard work of being a patient, which may have been exacerbated owing to having had a comparatively unusual surgery. Previous studies report that patients with rare diseases are often forced to become more knowledgeable and self-directed in their healthcare utilisation and help-seeking,^38^ despite the fact that those with severe illness (including pancreatic cancer) frequently prefer the physician to initiate and dominate decisionmaking conversations.^5,39^ Participants in the present study reflected that when the GP had been proactive, they felt more confident initiating further consultations, demonstrating that help-seeking is often recursively dependent on previous experiences of health services.^40^

On average, GP practices are increasing in size and there is evidence to suggest that larger practices provide higher quality care; for example, larger practices have, on average, higher Quality and Outcomes Framework (QOF) scores and fewer avoidable emergency admissions.^41^ However, smaller practices achieve higher QOF scores for patient experience, perhaps because of a greater degree of continuity of care with a familiar GP.^41^ The participants in the present study who did receive support from their practice reported that it was usually provided by the same GP. This offers a parallel to the patient seeing the same clinical team at hospital appointments and allows a stronger therapeutic relationship to be forged between doctor and patient. However, with general practices increasing in size over time, people with unusual conditions may find it increasingly difficult to seek help from a GP who is familiar with them and their medical history.

Participants also highlighted the need for their family to receive emotional support. Family members may take on the role of ‘enlisted carer’ without sufficient support or knowledge of what to expect, and, as patients themselves do, they may also hide their anxieties and present a positive appearance to the person with cancer.^42^ While family members can accompany the patient to hospital appointments, surgeons and oncologists do not have clinical responsibility towards them. On the other hand, family members may be registered with the same general practice, offering a unique opportunity for primary care to provide support to both the patient and their relatives.

It has been shown previously that although GPs feel that they are best placed to initiate and coordinate care for patients living with and beyond cancer, they feel that they lack the time, resources, and knowledge.^27^ As a result, GPs may revert to a reactive rather than a proactive attitude towards patients with cancer, relying on the patient to contact the practice to engage in transactional care.^43,44^ ‘Cancer care review’ models may be beneficial but could lack the holistic approach of a more open discussion between the GP and the patient and/or their family about the impact of cancer.^21^

Instead of a transactional approach to patients with long-term conditions, relationship-based care may be more appropriate and has been highlighted as a priority by the Royal College of General Practitioners. This describes care in which process and outcomes are enhanced by a high quality therapeutic relationship often developed over time. However, previous interactions between doctor and patient are not mandatory and a trusting, compassionate relationship can be built without this. Successful relationship-based care, which incorporates continuity of care as the relationship develops, may lead to better patient outcomes, including greater patient satisfaction, lower mortality, and reduced healthcare costs.^45–48^ It may also increase GP job satisfaction.^49^

In addition to the burden of uncertainty when help-seeking, patients with chronic illness also experience burden from the treatment itself. This may change over time and include physical side effects, attending appointments, managing inconvenient and restrictive treatment regimens, and dealing with interference to day-to-day life.^50^ Self-managing a long-term condition with complex medication regimens can be overwhelming for patients and caregivers, and may require high levels of knowledge and skill.^51^ This could offer an opportunity for other primary care staff, such as community pharmacists or practice nurses, to give ongoing support and education in this area, and could relieve some pressure from GPs.^52^

### Implications for research and practice

It is critical for GPs to recognise that some patients may not feel confident seeking support from primary care. Being proactive and asking about the psychological impact of living with and after cancer may mitigate this. It is important to recognise that patients may not be interested in support groups but may simply value someone to talk to without feeling ‘a burden’: this is the value of the relationship between clinician and patient in primary care. Other members of the primary care team, such as the pharmacist or nurse, may also be key in supporting patients in managing their medications. Flexibility should be enabled with prescriptions of PERT so that patients can easily access the necessary doses. Family members may be registered at the same GP practice, so the GP could identify their unmet support needs. Future research should consider direct exploration of family members’ needs, and the perspective of primary care practitioners about how the practice can support patients. Brief guidance for GPs about key issues faced by patients following pancreaticoduodenectomy could be created and its utility tested. This could be kept in the patient’s notes for ease of access. Support interventions that could be delivered in primary care should be developed and their acceptability and efficacy assessed.

## Data

Owing to the sensitive nature of the data and the potential for participants to be identified, the dataset is not publicly available.

## References

[1] Cancer Research UK Pancreatic cancer statistics. https://www.cancerresearchuk.org/health-professional/cancer-statistics/statistics-by-cancer-type/pancreatic-cancer.

[2] Rawla P, Sunkara T, Gaduputi V (2019). Epidemiology of pancreatic cancer: global trends, etiology and risk factors. World J Oncol.

[3] Cancer Research UK (2018). Chemotherapy, radiotherapy and tumour resections in England: 2013–14 V2. http://www.ncin.org.uk/publications/reports.

[4] Ziebland S, Chapple A, Evans J (2015). Barriers to shared decisions in the most serious of cancers: a qualitative study of patients with pancreatic cancer treated in the UK. Health Expect.

[5] Schildmann J, Ritter P, Salloch S (2013). 'One also needs a bit of trust in the doctor ... ': a qualitative interview study with pancreatic cancer patients about their perceptions and views on information and treatment decision-making. Ann Oncol.

[6] Deobald RG, Cheng ESW, Ko Y-J (2015). A qualitative study of patient and clinician attitudes regarding surveillance after a resection of pancreatic and peri-ampullary cancer. HPB.

[7] Blakely K, Karanicolas PJ, Wright FC, Gotlib Conn L (2017). Optimistic honesty: understanding surgeon and patient perspectives on hopeful communication in pancreatic cancer care. HPB.

[8] Evans J, Chapple A, Salisbury H (2014). "It can't be very important because it comes and goes"— patients' accounts of intermittent symptoms preceding a pancreatic cancer diagnosis: a qualitative study. BMJ Open.

[9] Mills K, Birt L, Emery JD (2017). Understanding symptom appraisal and help-seeking in people with symptoms suggestive of pancreatic cancer: a qualitative study. BMJ Open.

[10] Huang JJ, Yeo CJ, Sohn TA (2000). Quality of life and outcomes after pancreaticoduodenectomy. Ann Surg.

[11] Watson EK, Brett J, Hay H (2019). Experiences and supportive care needs of UK patients with pancreatic cancer: a cross-sectional questionnaire survey. BMJ Open.

[12] Dauphin S, Jansen L, De Burghgraeve T (2019). Long-term distress in older patients with cancer: a longitudinal cohort study. BJGP Open.

[13] Landers A, Brown H, Strother M (2019). The effectiveness of pancreatic enzyme replacement therapy for malabsorption in advanced pancreatic cancer, a pilot study. Palliat Care.

[14] Gooden HM, White KJ (2013). Pancreatic cancer and supportive care—pancreatic exocrine insufficiency negatively impacts on quality of life. Support Care Cancer.

[15] Labori KJ, Hjermstad MJ, Wester T (2006). Symptom profiles and palliative care in advanced pancreatic cancer: a prospective study. Support Care Cancer.

[16] Dengsø KE, Tjørnhøj-Thomsen T, Dalton SO (2018). Gut disruption impairs rehabilitation in patients curatively operated for pancreaticoduodenal cancer — a qualitative study. BMC Cancer.

[17] Guarner L, Rodríguez R, Guarner F, Malagelada JR (1993). Fate of oral enzymes in pancreatic insufficiency. Gut.

[18] Domínguez-Muñoz JE (2009). Pancreatic enzyme replacement therapy: exocrine pancreatic insufficiency after gastrointestinal surgery. HPB.

[19] Pancreatric Section, British Society of Gastroenterology, Pancreatic Society of Great Britain and Ireland, Association of Upper Gastrointestinal Surgeons of Great Britain and Ireland (2005). Guidelines for the management of patients with pancreatic cancer periampullary and ampullary carcinomas. Gut.

[20] National Institute for Health and Care Excellence (2018). Pancreatic cancer in adults: diagnosis and management. NICE guideline [NG85]. https://www.nice.org.uk/guidance/ng85/chapter/Recommendations#nutritional-management.

[21] Gopal DP, de Rooij BH, Ezendam NPM, Taylor SJC (2020). Delivering long-term cancer care in primary care. Br J Gen Pract.

[22] Noteboom EA, Perfors IA, May AM (2021). GP involvement after a cancer diagnosis; patients' call to improve decision support. BJGP Open.

[23] Wallner LP, Abrahamse P, Uppal JK (2016). Involvement of primary care physicians in the decision making and care of patients with breast cancer. J Clin Oncol.

[24] Wieldraaijer T, de Meij M, Zwaard S (2019). Introducing a time out consultation with the general practitioner between diagnosis and start of colorectal cancer treatment: patient-reported outcomes. Eur J Cancer Care.

[25] Brandenbarg D, Roorda C, Stadlander M (2017). Patients' views on general practitioners' role during treatment and follow-up of colorectal cancer: a qualitative study. Fam Pract.

[26] Anvik T, Holtedahl KA, Mikalsen H (2006). "When patients have cancer, they stop seeing me”—the role of the general practitioner in early follow-up of patients with cancer—a qualitative study. BMC Fam Pract.

[27] Walter FM, Usher-Smith JA, Yadlapalli S, Watson E (2015). Caring for people living with, and beyond, cancer: an online survey of GPs in England. Br J Gen Pract.

[28] Meiklejohn JA, Mimery A, Martin JH (2016). The role of the GP in follow-up cancer care: a systematic literature review. J Cancer Surviv.

[29] Bryman A (2004). Social Research Methods.

[30] Gilbert N (2008). Researching Social Life.

[31] O'Brien BC, Harris IB, Beckman TJ (2014). Standards for reporting qualitative research: a synthesis of recommendations. Acad Med.

[32] Braun V, Clarke V (2006). Using thematic analysis in psychology. Qual Res Psychol.

[33] Glaser BG (1965). The constant comparative method of qualitative analysis. Social Problems.

[34] Boeije H (2002). A purposeful approach to the constant comparative method in the analysis of qualitative interviews. Qual Quant.

[35] O’Connor MK, Netting FE, Thomas ML (2008). Grounded theory: managing the challenge for those facing institutional review board oversight. Qualitative Inquiry.

[36] Braun V, Clarke V (2021). To saturate or not to saturate? Questioning data saturation as a useful concept for thematic analysis and sample-size rationales. Qual Res Sport Exerc Health.

[37] Henwood KL, Pidgeon NF (1992). Qualitative research and psychological theorizing. Br J Psychol.

[38] Budych K, Helms TM, Schultz C (2012). How do patients with rare diseases experience the medical encounter? Exploring role behavior and its impact on patient-physician interaction. Health Policy.

[39] Ende J, Kazis L, Ash A, Moskowitz MA (1989). Measuring patients' desire for autonomy: decision making and information-seeking preferences among medical patients. J Gen Intern Med.

[40] Rogers A, Kennedy A, Nelson E, Robinson A (2005). Uncovering the limits of patient-centeredness: implementing a self-management trial for chronic illness. Qual Health Res.

[41] Kelly E, Stoye G (2014). Does GP practice size matter? GP practice size and the quality of primary care (Institute for Fiscal Studies). https://ifs.org.uk/publications/7445.

[42] Gerhardt S, Dengsø KE, Herling S, Thomsen T (2020). From bystander to enlisted carer — a qualitative study of the experiences of caregivers of patients attending follow-up after curative treatment for cancers in the pancreas, duodenum and bile duct. Eur J Oncol Nurs.

[43] Lawrence RA, McLoone JK, Wakefield CE, Cohn RJ (2016). Primary care physicians' perspectives of their role in cancer care: a systematic review. J Gen Intern Med.

[44] Salisbury H (2020). Is transactional care enough?. BMJ.

[45] Baker R, Streatfield J (1995). What type of general practice do patients prefer? Exploration of practice characteristics influencing patient satisfaction. Br J Gen Pract.

[46] Atlas SJ, Grant RW, Ferris TG (2009). Patient-physician connectedness and quality of primary care. Ann Intern Med.

[47] Pereira Gray DJ, Sidaway-Lee K, White E (2018). Continuity of care with doctors—a matter of life and death? A systematic review of continuity of care and mortality. BMJ Open.

[48] Hussey PS, Schneider EC, Rudin RS (2014). Continuity and the costs of care for chronic disease. JAMA Intern Med.

[49] Ridd M, Shaw A, Salisbury C (2006). 'Two sides of the coin’—the value of personal continuity to GPs: a qualitative interview study. Fam Pract.

[50] Sav A, King MA, Whitty JA (2015). Burden of treatment for chronic illness: a concept analysis and review of the literature. Health Expect.

[51] Mair FS, May CR (2014). Thinking about the burden of treatment. BMJ.

[52] Hindi AMK, Schafheutle EI, Jacobs S (2019). Community pharmacy integration within the primary care pathway for people with long-term conditions: a focus group study of patients', pharmacists' and GPs' experiences and expectations. BMC Fam Pract.

